# LESSONS LEARNED ANALYZING COMPLICATIONS AFTER LAPAROSCOPIC TOTAL GASTRECTOMY FOR GASTRIC CANCER

**DOI:** 10.1590/0102-672020200003e1539

**Published:** 2020-12-18

**Authors:** Italo BRAGHETTO, Enrique LANZARINI, Maher MUSLEH, Luis GUTIÉRREZ, Juan Carlos MOLINA, Owen KORN, Manuel FIGUEROA, Juan Pablo LASNIBAT, Omar ORELLANA

**Affiliations:** 1 Department of Surgery, Hospital José J. Aguirre, Faculty of Medicine, University of Chile, Santiago, Chile

**Keywords:** Stomach neoplasms, Laparoscopy, Gastrectomy, Neoplasias gástricas, Laparoscopia, Gastrectomia

## Abstract

**Background:**

Laparoscopic surgery has been gradually accepted as an option for the surgical treatment ofgastric cancer. There are still points that are controversial or situations that are eventually associated with intra-operative difficulties or postoperative complications.

**Aim:**

To establish the relationship between the difficulties during the execution of total gastrectomy and the occurrence of eventual postoperative complications.

**Method:**

The operative protocols and postoperative evolution of 74 patients operated for gastriccancer, who were subjected to laparoscopic total gastrectomy (inclusion criteria) were reviewed. The intraoperative difficulties recorded in the operative protocol and postoperative complications of a surgical nature wereanalyzed (inclusion criteria). Postoperative medical complications were excluded (exclusion criteria). For the discussion, an extensive bibliographical review was carried out.

**Results:**

Intra-operative difficulties or complications reported correspond to 33/74 and of these; 18 events (54.5%) were related to postoperative complications and six were absolutely unexpected. The more frequent were leaks of the anastomosis and leaks of the duodenal stump; however, other rare complications were observed. Seven were managed with conservative measures and 17 (22.9%) required surgical re-exploration, with a postoperative mortality of two patients (2.7%).

**Conclusion:**

We have learned that there are infrequent and unexpected complications; the treating team must be mindful of and, in front of suspicion of complications, anappropriate decision must be done which includes early re-exploration. Finally, after the experience reported, some complications should be avoided.

## Graphical Abstract

[Fig f1a]

**Figure f1a:**
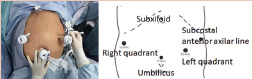
Grassi´s - French position for laparoscopic esophago-gastric procedures

## RESUMO

**Racional:**

A cirurgia laparoscópica tem sido gradualmente aceita como opção para o tratamento cirúrgico do câncer gástrico. Ainda existem pontos controversos ou situações eventualmente associadas a dificuldades intra-operatórias ou complicações pós-operatórias.

**Objetivo:**

Estabelecer a relação entre as dificuldades durante a execução da gastrectomia total e a ocorrência de eventuais complicações pós-operatórias.

**Método:**

Foram revisados ​​os protocolos operatórios e a evolução pós-operatória de 74 pacientes operados por câncer gástrico, submetidos à gastrectomia total laparoscópica (critérios de inclusão). Foram analisadas as dificuldades intraoperatórias registradas no protocolo operatório e as complicações pós-operatórias de natureza cirúrgica (critérios de inclusão). As complicações médicas pós-operatórias foram excluídas (critérios de exclusão). Para a discussão, foi realizada extensa revisão bibliográfica.

**Resultados:**

Dificuldades ou complicações intraoperatórias relatadas corresponderam a 33/74 e destas 18 (54,5%) foram relacionadas com complicações pós-operatórias e seis absolutamente inesperadas. As mais frequentes foram vazamentos da anastomose e do coto duodenal; no entretanto, outras complicações raras foram observadas. Sete foram tratados com medidas conservadoras e 17 (22,9%) necessitaram de re-exploração cirúrgica, com mortalidade pós-operatória de dois pacientes (2,7%).

**Conclusão:**

Aprendemos que existem complicações infrequentes e inesperadas; a equipe de tratamento deve estar atenta e diante da suspeita de complicação, decisão apropriada pode incluir uma nova exploração precoce. Finalmente, após a experiência relatada, algumas complicações devem ser evitadas.

## Graphical Abstract

[Fig f1as]

**Figure f1as:**
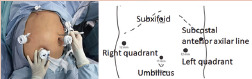
Posição de Grassi francesa para procedimentos esofagogástricos laparoscópicos

## INTRODUCTION

Laparoscopic surgery has been gradually accepted as an option for the surgical treatment of gastric cancer, first in Asian countries and then in Europe. It was initially accepted with hybrid procedures for subtotal gastrectomies and then total gastrectomies, which is the procedure that represents the greatest challenges. Laparoscopic surgery has proved to be safe and effective with less pain, less bleeding and a shorter recovery time. The randomized studies during the last decade have shown excellent results in terms of complication rates, which are very similar to open surgery^1^ ^,^ ^2^ ^,^ ^20^ ^,^ ^21^ ^,^ ^28^ ^,^ ^29^.

In terms of oncological safety, the number of lymph nodes and resected lymph nodal barriers do not differ in any way from open surgery^1^ ^,^ ^24^. Moreover, it is possible that lymph node dissection becomes more precise and less difficult with 3D laparoscopic or robotic surgery (R0 oncological surgery with better survival)^22^ ^,^ ^24^ ^,^ ^30^.

However, there are still points that are under discussion concerning the surgical technique itself, such as: 1) type of lymphadenectomy; 2) bursectomy: yes or no?; 3) total or partial major omentectomy; 4) management of the duodenal stump; 5) type of esophagojejunal anastomosis; 6) jejunojejunostomy; 7) extraction of the stomach and omentum. These are the most controversial points or situations that are eventually associated with intraoperative difficulties or postoperative complications^5^ ^,^ ^21^ ^,^ ^22^ ^,^ ^27^.

The objective of this article was to establish the relationship between the difficulties during the execution of total gastrectomy and the occurrence eventual postoperative complications. Analysis of the experience gathered by our work team can be useful for the prevention of these complications.

## METHOD

### Ethical statements

This article does not contain experimental human studies. All procedures performed were in accordance with the ethical standards of the responsible committee of our institution and the operation was conducted in accordance with the Helsinki Declaration. This study was approved by the Ethic Committee of Research of our Hospital. The official informed consent form used in our hospital was obtained and signed from all patients before the operation.

We reviewed the prospective registry of the oncologic statistical unit of our Department. The operative protocols and postoperative evolution of patients operated for gastric cancer, who were subjected to a laparoscopic total gastrectomy (inclusion criteria) by our team between January 2010 and December 2018, were reviewed. Seventy four patients whose demographic characteristics are presented in Table 1 were included for this analysis.

**FIGURE 1:**
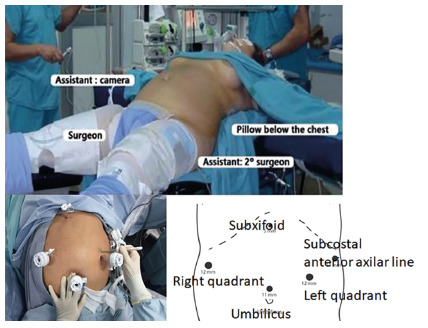
Grassi´s - French position for laparoscopic esophago-gastric procedures

**FIGURE 2:**
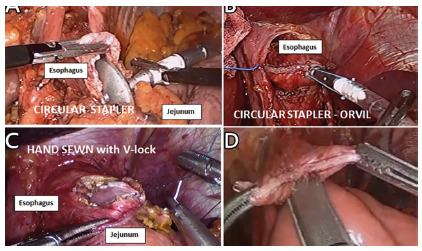
Types of esophago-jejuno anastomosis performed withcircular stapler (n=13), OrVil® system(n=3), linear stapler (n=44) and hand sewn running suture (n=14): A) circular stapler placement opening the distal esophagus; B) Orvil® system placed by oral route and pulled down by laparoscopic route; C) manual esophago-jejunum anastomosis using running V-lock suture; D) linear stapler placement opening the distal esophageal stump and jejunal wall for laterolateral esophago-jejunal anastomosis.

The basic steps for total laparoscopic gastrectomy were: 1) French Grassi´s position with 5 work´s ports (Figure 1); 2) R0 resection (but R1 resections should be considered in cases palliative treatment of neoplastic complications); 3) lymphadenectomy D1 (+) or D2 (mean 29 nodes); 4) esophagojejunal anastomosis. We have used running hand sewn with 000 V-Lock^®^ suture, with circular stapler, withOrVilTM system or with linear stapler, (Medronic, Mansfield, MA, USA, Figure 2)

**TABLE 1:** Demographic characteristics of patients undergoing laparoscopic gastrectomy

Age: mean 69.1 years (range 25-87 years)		
Sex: male 46, female 28		
Histological type: adenocarcinoma	n	%
Well differentiated	9	12.2
Moderately differentiated	17	22.9
Poorly differentiated	28	37.8
Seal ring cells	15	20.3
Neuroendocrine tumor	5	6.8
Location:		
Upper third	30	40.5
Middle third	28	37.8
Lower third	11	14.9
Diffuse multifocal	5	6.8
Stage:		
0	4	5.4
Ia	15	20.3
Ib	5	6.8
IIa	9	12.2
IIb	9	12.2
IIIa	8	10.8
IIIb	6	8.1
IIIc	8	10.8
IV	4	5.4

The intraoperative difficulties recorded in the operative protocol and postoperative complications of a surgical nature (inclusion criteria) were analyzed. Postoperative medical complications were excluded (exclusion criteria). 

For the discussion, an extensive bibliographical review was carried out on the seven points that motivated the discussion and that were subsequently raised in the introduction.

## RESULTS

Our team has operated on 74 gastric cancer patients who were subjected to a total laparoscopic gastrectomy (inclusion criteria). Patients undergoing distal subtotal gastrectomy and segmental gastric resections were not included in this analysis (exclusion criteria). The average operating time was 273 min (215-478). No major losses of blood were reported and there were no reports of a need for red blood cell transfusions. Table 2 shows the details of surgical techniques that are analyzed in this work. Conversion to open surgery occurred in one patient with a large tumor adhered to the greater omentum towards the hepatic angle of the colon, which was possible to completely release. The intracorporeal anastomoses were made, but at the time of extraction, a 10 cm supraumbilical midline laparotomy had to be performed to extract the stomach and omentum.

### The intraoperative difficulties

Management to solve them and their relationship with postoperative complications are indicated in Table 3. At the time of the group 6 lymphnode dissection, problems arose in two patients. In one patient, the ultrasonic scalpel perforated the medial aspect of the duodenal wall and, therefore, a duodenorrhaphy was necessary. In a second patient with a large ganglionic conglomerate, a very low duodenal dissection was needed in the second portion of the duodenum. These two patients presented a duodenal fistula in the postoperative period. Bleeding occurred during the lymphadenectomy in 10 cases which required the use of energy for hemostasis or placement of Surgicel^®^ in both retroperitoneal and splenic hilus were related with postoperative hemoperitoneum in one and three splenic necrosis. During the bursectomy (n=13), the dissection layer was not easy to recognize in two patients. In one, ultrasonic scalpel damage of the anterior side of the pancreatic parenchyma occurred. In another, with neoplasic involvement of the anterior face of the pancreas, a distal pancreatectomy was performed. Both evolved to acute postoperative pancreatitis and pancreatic fistula that were difficult to manage. In other cases, a bursectomy was simply not performed due to the difficulties in identifying the correct plane of cleavage.

**TABLE 2:** Details of the surgical technique performed

		n	%
1. - Lymphadenectomy	D1+	15	20.3
	Dl	5	6.8
	D2	51	68.9
	D2 ext.	3	4.1
2. - Bursectomy	Consigned	13	17.5
3. - Omentectomy	Total	65	87.8
	Partial	9	12.2
4. - Closure of duodenal stump linear stapler	Without reinforcement	73	98.6
	With reinforcement	1	1.4
5. - Esophagojejunal anastomosis	Linear	44	59.5
	Manual	14	18.9
	Circular	13	17.6
	Orvil® system	3	4.0
	Methylene blue test positive	6	8.1
	negative	46	62.2
	not consigned	12	16.2
	Suture reinforcement	37	50.0
6. - Jejuno-jejunal anastomosis	Linear stapler with angle reinforcement	73	98.7
	Without reinforcement	1	1.3
7. - Extraction of stomach	Pfannenstiel	58	78.4
	Umbilical	15	20.3
	Converted	1	1.3

### During omentectomy

In one of our cases it was very difficult to lift the greater omentum even with the combined maneuvers of the surgeon and assistant which was probably the cause of a tear and subsequent intestinal perforation 3 cm from the duodeno-jejunal (Treitz) angle detected postoperatively that resulted in an urgent re-laparoscopy as it rapidly evolved to diffuse peritonitis. In a second one case a partial omentectomy and adherensiolysis was performed, intestinal obstruction with loop necrosis occurred that led to its resection. Another third patient, who required the resection of a large omental implant, was subjected to major laparotomy for its extraction and was catalogued as conversion. During duodenal stump management, in two patients (already mentioned in the previous paragraph) a dissection was performed at the second portion to ensure a limit of distal section free of neoplastic invasion (>1 cm). The mechanical suture line was not reinforced which subsequently led to a duodenal fistula. In two other patients, periduodenal bleeding occurred without major repercussions.

**TABLE 3:** Intraoperative events and their relationship with postoperative complications

Surgical moment	Intraoperative event	Postoperative complication
Lymph node dissection	Duodenal wall injury (n=2) Bleeding splenic hilum (n=1) Bleeding periduodenal (n=3) Retroperitoneal (n=1)	Duodenal stump leak (n=2)* Splenic necrosis (n=1) Hemoperitoneum (n=3)
Bursectomy	Pancreatic damage (n=2)	Pancreatic leaks (n=2)
Omentectomy	Difficult mobilization (n=4) Adhesions (n=1) Omental implant (n=1)	Bowel perforation (n=1) Bowel obstruction (n=1) Bowel necrosis (n=1) Conversion (n=1)
Duodenal stump	Difficult dissection (n=2) Periduodenal bleeding (n=2)*	Stump leak (n=2)*|
Esophagojejunostomy	Blue methylene test (+) (n = 6) Anastomotic leak (n=6) eliminar Re-resection (n=3)* Tension anastomosis (n=1)	Anastomotic leak (n=6)
Jejunojejunostomy	Suture leakage (n = 1)	Postoperative leak (n=1)
Stomach extraction	Wound contamination (n=1) Large tumor/omental implant	Necrotizing fasciitis (n=1) Conversion*

**same patient

In our group, different types of esophago-jejunal anastomosis have been performed, being a significant number with latero-lateral anastomosis linear stapler, few cases with latero-terminal anastomosis with a conventional circular stapler, Orvil^®^ system or manual anastomosis (Figure 2). When conducting the methylene blue test, leakage was observed in six patients. This motivated reinforcement with separate sutures which is also difficult to execute. In the postoperative period, fistula of the esophago-jejunal anastomosis was observed in these six patients, probably related to these difficulties.

### Jejunojejunostomy

In one patient without reinforced suture of stapled line presented leakage of the anastomosis despite the negative methylene blue test. Early bile fluid through the drainage was detected, reoperation was immediately performed and the patient progressed very well.

### Extraction of the stomach and omentum

In one patient there was difficulty extracting the tissue sample through a suprapubic Pfannenstiel´s incision in the skin fold due to its large size. Probable poor skin cleaning resulted in a severe infection of the operative wound and necrotizing fasciitis. In another patient with omental infiltration (already commented), a larger laparotomy had to be performed and it was considered as a conversion. The sum of intraoperative difficulties or complications reported correspond to 33/74 and of these 18 (54.5%) were related to postoperative complications. Therefore, surgical management during the procedure was successful in 15 patients.

### Postoperative complications

Table 4 shows the major postoperative complications. A total of 24 complications were observed (32.4%), some of them were directly related to the intraoperative difficulties already described (n=18, 75%), but six (25%) were absolutely unexpected. The most frequent were leaks of the anastomosis and leaks of the duodenal stump. Seven were managed with conservative measures and 17 (22.9%) required surgical re-exploration, with a postoperative mortality of two patients (2.7%), the first one due to leak of esophago-jejunostomy and the second due to severe necrotizing fasciitis. The observed pancreatic leaks were directly related to intraoperative complications during the bursectomy. Both patients were re-operated and finally developed well, but their hospital stay was very long. Hemoperitoneum was observed in three. A cautionary note: It is difficult to prescribe anticoagulant treatment in the postoperative period since on the one hand there is the risk of hemorrhage and on the other the risk of thromboembolism. Aggressive necrotizing fasciitis occurred in one case that died of septic shock 45 days after the operation despite periodic surgical toilets. For the intestinal perforation close to the duodeno-jejunal angle due to intraoperative undiagnosed jejunal tear, was early re-operated performing jejunorrhaphy; however, the patient evolved with intraperitoneal collections that improved with further surgical peritoneal cleaning. The other patient presented an intestinal ischemia that had to be re-operated for intestinal resection, with satisfactory outcome. 

**TABLE 4:** Postoperative surgical complications after laparoscopic total gastrectomy for gastric cancer (n=74)

		Reoperation	Mortality
	n	n	n
Esophagojejunal anastomosis fistula	6	3	1
Duodenal stump fistula	5	3	
Pancreatic fistula	2	2	
Hemoperitoneum	3	3	
Intestinal perforation	2	2	
Intestinal obstruction	2	2	
Subphrenic abscess	2		
Jejuno-jejunal anastomosis leakage	1	1	
Necrotizing fasciitis	1	1	1
Total	24 (32.4%)		
Reoperation		17 (22.9%)	
Operative mortality			2 (2.7%)

## DISCUSSION

The laparoscopic approach has been gaining prestige in the last few years, since it is perfectly possible to completely resect R0 in a safe manner. It will probably become the technique of choice in the future, provided that the selection criteria for each appropriate procedure are respected for each patient. Multiple studies have demonstrated the effectiveness and oncological results of the laparoscopic technique. In a recent review by Son et al.^28^, cumulative results from multiple trials showed no significant difference in terms of survival rate or recurrence between open or laparoscopic surgery. Many studies have shown that the number of harvested lymph nodes was equivalent in both groups. In patients with large T4 tumors, laparoscopic surgery is not justified and open surgery still has its place (multi-visceral resection) in spite of having undergone neo-adjuvance that require extended resections. However, conversion therapy for gastric cancer has been proposed^5^ ^,^ ^8^ ^,^ ^20^ ^,^ ^24^ ^,^ ^25^ ^,^ ^29^ ^,^ ^30^. We made an analysis based on the difficulties and postoperative complications observed by our team that is similar to those reported by other national and foreign groups.

### Lymph node dissection

**FIGURE 3:**
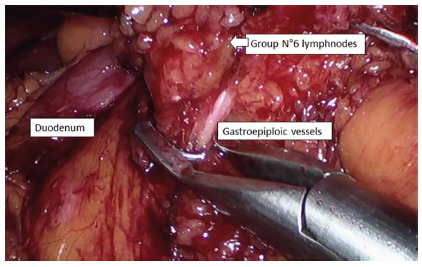
Group 6 lymph node dissection: fatty tissue and lymph nodes are dissected and lifted sectioning at the base of gastroepiploic vessels

We have always followed the Japanese school for D2 lymph node dissection, which has globally been accepted^3^. There are no difficulties in lymph node dissection in general^5^. Group 6 lymph node dissection probably presents the most difficulties. For the expeditious management of the dissection of this group, especially when there are large lymph node conglomerates, we suggest that the separation of the anterior leaf from the meso of the transverse colon be continued until the medial wall of the second portion of the duodenum. From there, begin dissection and lift the fatty tissue until the emergency of the gastroepiploic vessels which must be dissected and clipped separately. With this maneuver, we think that the difficulties and the risks of complication, such as an injury to the pancreatic tissue or the duodenal wall, are minimized (Figure 3). 

**FIGURE 4:**
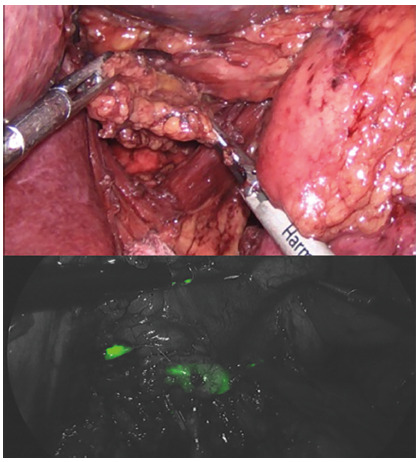
Laparoscopic lymph node dissection: image with and without indocyanine green

In groups 8, 7 and 9 the dissection did not present great difficulties, aside from annoying bleeding. For group 12 dissection, special care had to be taken during the lymphadenectomy in order to avoid damage to the hepatic pedicle or the portal vein. For the group 11d lymphadenectomy, we must dissect the superior border of pancreas and if it is anatomically possible to resect those of group 10 (splenic hilum) without increasing the risk of complications such as bleeding or pancreatic fistula. In laparoscopic surgery, we have used 3D optical systems and our impression is that lymph node dissection is safer and even more with robotics^5^ ^,^ ^19^ ^,^ ^23^. Another advance is the use of indocyanine green injection for better identification of infiltrated lymph nodes^6,23^ (Figure 4).

### Bursectomy

This is a highly controversial critical point, since in some cases it is easyand the pneumoperitoneum helps in the identification of the adequate layer of dissection; however, in other cases, there is neoplastic or desmoplastic infiltration, which makes its dissection difficult. In the 1960s, for Japanese gastric cancer society, the bursectomy was constituted as a fundamental element in the radical surgical management of gastric cancer, for diminished the local recurrence of the disease^7^ ^,^ ^23^ ^,^ ^26^. However, more recent data confirm that gastrectomy with bursectomy is not superior to non-bursectomy in terms of survival. Bursectomy is not recommended as a routine procedure for the surgical treatment of gastric cancer^25^. Currently, the tendency is to perform it only in case of obvious infiltration.

### Omentectomy

Although it is not a critical step within the procedure of a gastrectomy and there should be no major difficulties, there are cases in which the greater omentum is very thick and heavy and its mobilization becomes laborious and the discussion arises whether to perform partial or total omental resection. The greater omentum in laparoscopic surgery is generally conserved in a partial manner for various reasons: immunological, better handling of the piece, decreased operative time since and because there are no ganglia beyond 3-4 cm of the gastroepiploic arch. Recurrence free survival at 3-5 years is similar with a total or partial omentectomy, suggesting that gastrectomy with preservation of the omentum performed even in patients with advanced gastric cancer does not increase peritoneal recurrence or affect the survival of patients when compared to conventional gastrectomy. In the Japanese treatment guidelines for gastric cancer published in 2017, there are no definite comments regarding the omentectomy; however, these establish that the extirpation of the greater omentum is generally integrated in the standard gastrectomy for T3 (subserosa) or deeper tumors^6^. It seems prudent to wait for the results of studies with better methodological design before abandoning the practice of total omentectomy, especially in gastric cancer with compromise of the serosa^4^ ^,^ ^9^ ^,^ ^15^.

### Duodenal stump management

#### With stapler and reinforcement, yes or no?

Duodenal fistula post total gastrectomy, although rare (2%), is one of the complications that may lead to serious repercussions and even mortality (up to 15%) if it is not prevented, no early diagnosis or if no adequate treatment is carried out. Several studies support reinforcement. In our opinion, it depends on the duodenal stump characteristics. At the level of the second portion, the duodenal wall is thinner and the staples do not perform satisfactorily. We believe that in these cases, a reinforcing suture is recommended^11^ ^,^ ^17^ ^,^ ^18^.

### Esophagojejunal anastomosis.

For the esophagojejunal anastomosis, different modalities have been reported. All have their advantages and disadvantages and there is no scientific evidence to determine which esophago-jejunostomy technique is the best. Linear latero-lateral esophago-jejunostomy is quick and very comfortable in low intra-abdominal anastomoses, but it is not free of difficulties in high intramediastinal anastomoses since the visualization of the upper end of the suture is not optimal. It should always be reinforced since leaks are frequently detected which are also difficult. Termino-lateral anastomosis with the Orvil^®^ system used in a few patients is very elegant and safe but it is cumbersome to pass the system orally and introduce the stapler handle via one of the trocars, which must be widened. Its introduction into the intestinal lumen is not clean and intestinal wall tears frequently occur^12^. The circular stapler anastomosis has the same drawback although there are several “tricks” for the placement of the anvil; however, these maneuvers need training to perform them. Complications of esopha-gojejunal anastomosis occur between 5-10%, 2-4% correspond to fistulas and 1-9% stenosis. However, almost all the studies have reported that morbidity (such as leakage and anastomotic stenosis) for the two methods is not significantly different^10^ ^,^ ^12^ ^,^ ^13^ ^,^ ^14^ ^,^ ^16^ ^,^ ^23^ ^,^ ^28^ ^-^ ^32^. Few studies refer to the manual suture, which we consider very safe although it is slower and more laborious especially when the anastomosis is higher in the lower mediastinum. To ensure the inclusion of the esophageal mucosa completely and circumferentially, it is advisable to start the suture with two independent strands suture. An important aspect to point out occurs in anastomoses located in the inferior mediastinum that may be under stress. A recommendable maneuver is to lengthen the loop to be anastomosed by sectioning a vascular arcade so the end of the loop can reach higher without tension. We perform an esophago-jejunostomy with manual suture over a bougie in order to avoid strictures. 

### Jejunojejuno anastomosis

Few ones have been reported concerning jejuno-jejunal anastomosis, which usually does not present great difficulties; however, the end of the mechanical suture should always be reinforced since it is a point of risk of leakage. Avoid kinking or twist is mandatory.

### Extraction of the stomach and omentum

We have performed a suprapubic incision or a peri-umbilical incision. The difficulty arises when a very large piece with a large tumor or omentum must be removed. A suprapubic incision is particularly uncomfortable to close and although a periumbilical incision provides faster closure and better visualization of the planes, it could lead to an incisional hernia in the future.

The limitation of this study is because is a retrospective non-randomized study, but is a contribution to the knowledge for the management of intra and postoperative complications that can occur during and after laparoscopic total gastrectomy.

## CONCLUSION

We have learned that there are infrequent and unexpected complications that treating team must be mindful of, and when faced with the least suspicion of a complication, an appropriate decision which includes early re-exploration. Finally, after the experience reported, some complications should be avoided. 
